# Sporadic Creutzfeldt-Jakob Disease: Case Report and Literature Review

**DOI:** 10.7759/cureus.7767

**Published:** 2020-04-21

**Authors:** Asia Filatov, Javed L Khanni, Kettia Alusma-Hibbert, Patricio S Espinosa

**Affiliations:** 1 Neurology, Charles E. Schmidt College of Medicine, Florida Atlantic University, Boca Raton, USA; 2 Neurology, Boca Raton Regional Hospital, Boca Raton, USA; 3 Neurology, Marcus Neuroscience Institute, Boca Raton Regional Hospital, Boca Raton, USA

**Keywords:** creutzfeldt–jakob disease, cjd, scjd, subacute spongiform encephalopathy, prion disease, neurodegenerative, parkinson, parkinsonism

## Abstract

The proverbial “zebras” in neurology are often times missed due to their low prevalence and incidence in the community. The number of misdiagnoses and improper therapeutic interventions that occur are further increased when patients with these rare diseases present with signs and symptoms of more common disorders. One such disease is sporadic Creutzfeldt-Jakob disease (sCJD), a prion disease that causes neuronal derangement and classically presents as a rapidly progressing dementia with extrapyramidal signs, ataxia, behavioural problems, and myoclonus in the advanced stage. It falls into the category of neurodegenerative disease, which also includes Alzheimer's disease, Huntington's disease, Parkinson's disease, and other Parkinson-related diseases. Though these diseases have overlapping symptomologies - such as cognitive impairment and neuromuscular dysfunction - they can be differentiated from one another based on the time course of the illness and the specific constellation of signs and symptoms. Our case report describes a patient who was found to have sCJD after months of treatment for Parkinson's disease and trigeminal neuralgia. Thus, we are highlighting the importance of recognizing rare diseases so that proper management can be initiated in a timely manner. Furthermore, we review the current literature on the diagnosis and management of sCJD.

## Introduction

Creutzfeldt-Jacob disease (CJD) is a neurodegenerative disease that occurs following hereditary or sporadic mutations of the prion protein-encoding gene. Types of CJD include sporadic CJD (sCJD) (most common), familial, variant, and iatrogenic CJD. CJD is characterised by a build-up of protease-resistant prion proteins caused by a genetic polymorphism at number 129 amino acid residue, which can be either valine or methionine [1]. The purpose of this report is to present a rather unique case of a patient found to have sCJD and review the current published evidence on treatment options and management for CJD.

## Case presentation

**Figure 1:**
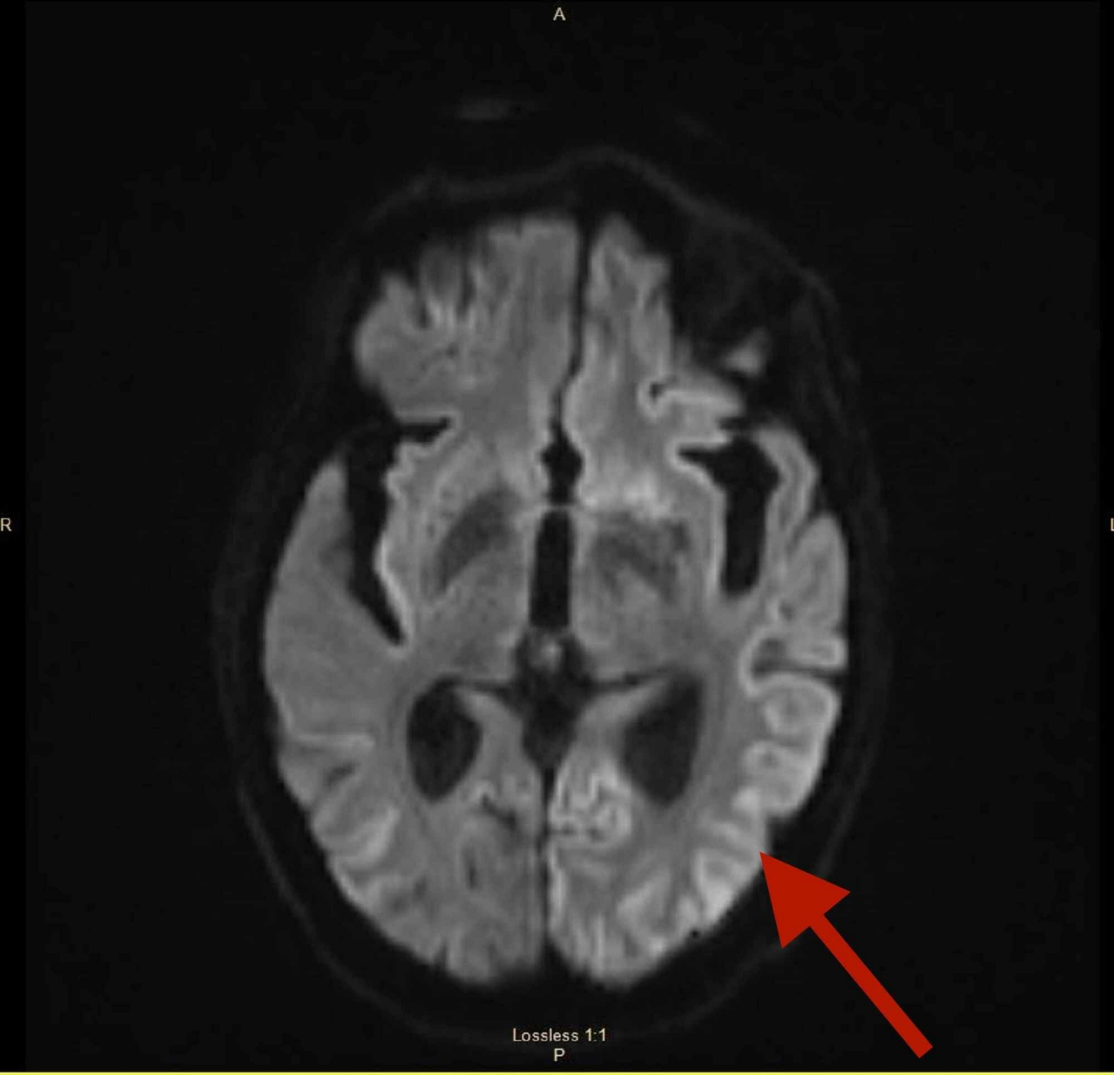
MRI brain: DWI axial image showed the ribbon-like signal hyperintensity of cerebral cortical gyri, which is known as the cortical ribbon sign DWI, diffusion-weighted imaging

Our patient was a 79-year-old female who presented with a six-month history of increased forgetfulness, muscle weakness, arm tremors, and changes in speech and penmanship. Her past medical history was significant for cerebellopontine angle vestibular schwannoma diagnosed four years ago and treated with CyberKnife therapy (Accuray Inc., Sunnyvale, CA) without complications or reoccurrence. After treatment, the patient did not develop any auditory deficits such as hearing loss or tinnitus consistent with persistent disease. Furthermore, she did not develop facial palsy following CyberKnife therapy. On physical examination, our patient was noted to be wheelchair-bound for locomotion. Mental status exam revealed moderate cognitive impairment with hesitation initiating speech/minimal speech, hypomania and hypophonia. Remaining neuro exam revealed facial asymmetry with voluntary movement, increased muscle rigidity, cogwheel rigidity, proximal muscle weakness in lower extremities (exemplified by difficulty getting from a sitting to a standing position), mild, bilateral upper extremity tremor, myoclonus and hesitation in gait initiation. MRI brain was conducted and revealed the ribbon-like signal hyperintensity of cerebral cortical gyri (cortical ribboning) in the parietal and occipital regions, known as the cortical ribbon sign, suggestive of CJD (Figure 1). 

Prior to presenting to our facility, this patient had been diagnosed with and treated for Parkinson's disease and trigeminal neuralgia without improvement. Due to the rapid progression of her disease, severity of her symptoms and failed treatment attempts over the last six months, we performed a diagnostic lumbar puncture to test for CJD. Cerebrospinal fluid (CSF) analysis at this time detected 14-3-3 proteins and the diagnosis of sCJD was made. Based on her clinical picture, it was determined that our patient had advanced stage sCJD. Therefore, she was admitted for close medical supervision and treatment. Our patient was only offered symptomatic management and supportive treatment due to the absence of standard therapy to date for advanced sCJD.

## Discussion

Patient case

For our patient, the road to sCJD diagnosis was shrouded with red herrings, misdiagnoses, and inappropriate treatment. Unfortunately for our patient, these treatments may have inadvertently hastened the progression of sCJD. Parkinson's disease typically presents with signs and symptoms such as bradykinesia, tremor at rest and akinesia [2]. It may also manifest with signs of postural disturbances and rigidity [3]. The use of carbidopa-levodopa in the initial management of motor symptoms may have been informed by the physical presentation of the disease that matched that of Parkinson’s disease. However, carbidopa-levodopa is associated with levodopa-induced dyskinesias, which may have occurred in our patient [4]. Another issue was the administration of Botulinum Toxin therapy for the management of trigeminal neuralgia. Botulinum Toxin therapy seemed to have complicated her condition with side effects such as regional weakness, edema at the site of injection, and facial asymmetry during voluntary movement [5].

**Figure 2:**
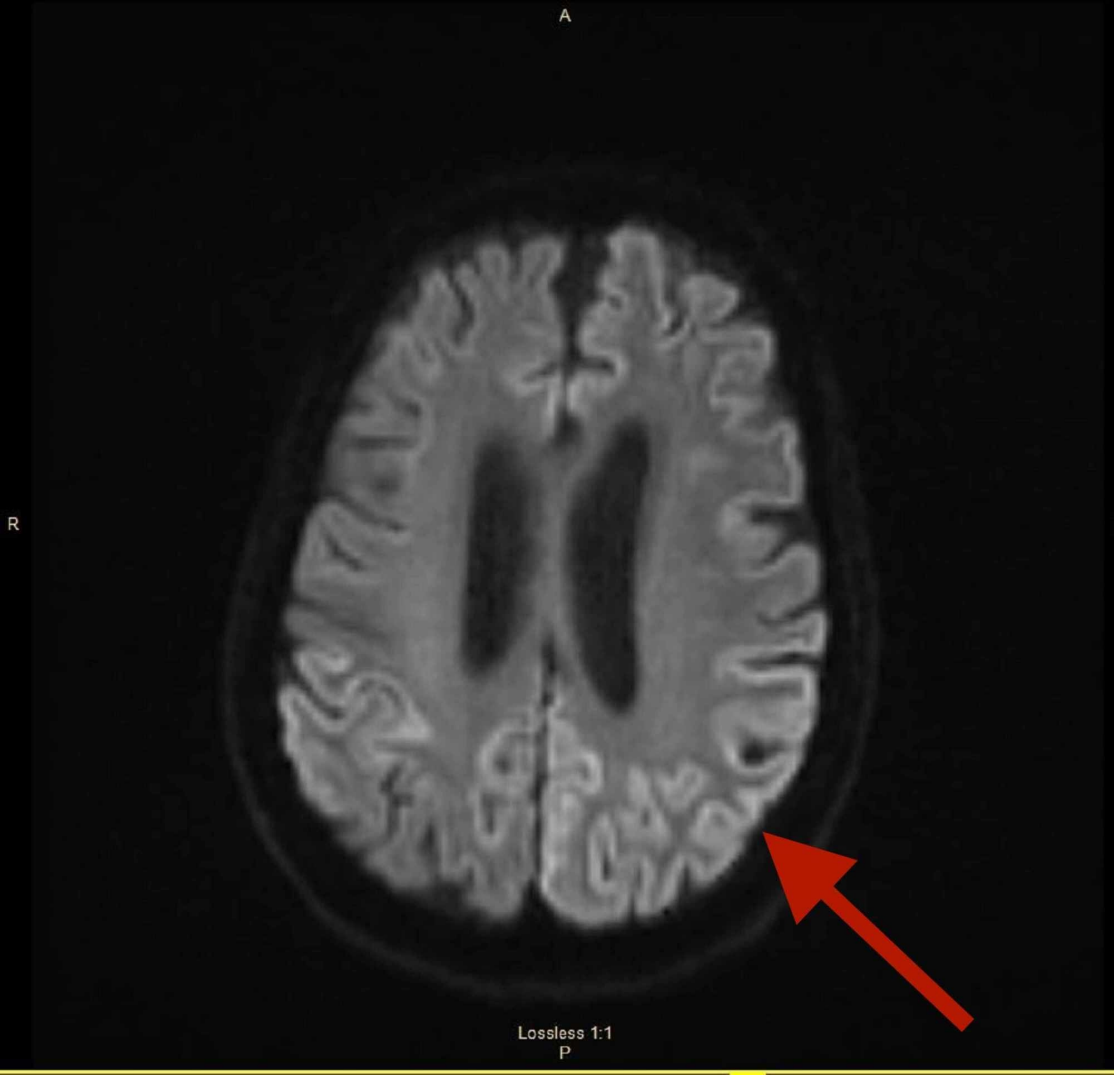
MRI brain shows a signal hyperintensity of cortical gyri, characteristically known as the cortical ribbon sign

Furthermore, with her barrage of symptoms, there was thought as to whether or not her current symptoms were any at all related to her history of cerebellopontine angle vestibular schwannoma and/or treatment with CyberKnife therapy. The primary goal of CyberKnife therapy is functional preservation [6]. Post-treatment outcome of vestibular schwannoma, by CyberKnife therapy, was positive in our patient since there was functional preservation immediately following treatment. Our patient showed preservation of hearing function, no tinnitus, no trigeminal neuropathy, and no facial palsy consistent with her condition/treatment [7]. In addition, known risks of CyberKnife surgery were not observed. These risks include CSF leak, hearing loss, headache, meningitis, and anaesthesia-associated complications [8]. Therefore, we came to the conclusion that there was no association between the current case of sCJD and her history of cerebellopontine angle vestibular schwannoma four years ago. MRI brain was an additional diagnostic imaging to rule in sCJD (Figure 2). 

All in all, we believe that earlier misdiagnoses and treatment may have hastened the progression of sCJD. Therefore, we emphasize that correct diagnosis is necessary for better treatment outcomes.

Pathophysiology of sCJD

CJD develops due to the accumulation of a misfolded prion protein and it is composed of four subtypes. The four subtypes of CJD include sporadic, familial, variant, and iatrogenic CJD. sCJD is associated with 85% of all cases of CJD and, therefore, is the most common [9]. Similarly, sCJD is the subject of this discussion. The classic presentation of sCJD is a rapid progressing dementia with extrapyramidal signs, ataxia, myoclonus in the advanced stage, and behavioural problems. In 35% of cases, cognitive decline is the key initial clinical presentation [9]. sCJD leads to a rapid cognitive decline, which explains why the patient, in this case, presented with memory loss, hesitation in the initiation of gait and speech, worsening penmanship, minimal speech, hypomania, and hypophonia. Sensory, motor, and visual functions are infrequently affected in the early stage of the disease [9]. However, this case was an advanced stage of the disease since the patient presented with motor and sensory complications such as myoclonus, tremors in bilateral upper extremities, increased muscle rigidity, hesitation in initiating gait and speech, minimal speech, cogwheel rigidity, and dependence on the wheelchair for movement.

Signs and symptoms of sCJD

The peak age of onset of sCJD is usually between 60 and 80 years of age [6]. Similarly, our patient presented in this case was 79 years old; thus, she fell within the age bracket for the onset of the disease. The vital clinical signs and symptoms of sCJD include progressive dementia, psychiatric manifestations, cognitive decline, and cerebellar ataxia. Dementia presents with multi-axial involvement of the nervous system [6]. Status epilepticus and seizures are uncommon findings in sCJD [10]. Furthermore, in sCJD progressive loss of cognitive and neurological functions takes a matter of weeks or a month; whereas similar changes associated with dementia take more than six months [11]. However, caution must be observed when making the diagnosis of sCJD since toxic encephalopathies may mimic sCJD in clinical presentation [12]. The patient, in this case, presented with similar signs and symptoms mentioned above, such as a decline in memory, poor motion and muscle coordination, hypophonia, tremor, and hypomania that rapidly progressed over a few months.

Treatment and management of sCJD

The mean survival of sCJD is six months; thus, the patient needed immediate and close treatment and care [6]. Due to the advanced stage of the disease, the patient was admitted for close medical attention and monitoring. No tentative discharge plan was made at the time of admission since the disease was expected to progress and worsen within a short period of time, indicating an increased dependence on hospital care [9]. However, the prognosis of the disease remains grim. The current aim of treatment is the provision of supportive care and symptomatic management to make the patient as comfortable as possible. To that end, our patient was administered morphine for relief of sCJD-associated pain and clonazepam for the management of myoclonus [13].

## Conclusions

Our patient represents an atypical clinical presentation of sCJD with regards to the age of onset, presenting signs and symptoms, and the rate at which symptoms progressed. Her presenting symptoms included progressive memory loss for the past six months, increased forgetfulness, muscle weakness, mild bilateral upper extremity tremor, and difficulty getting from a sitting to a standing position, which were indicative of sCJD. Positive diagnosis of sCJD was supported by the detection of 14-3-3 proteins in CSF. The disease was in an advanced stage at presentation and due to the aggressive and progressive nature of the disease, our patient was admitted for close medical care. Unfortunately, there is no standard, effective therapy for sJCD; hence, the patient was only offered supportive and symptomatic management. We hope that as more cases like this are studied, CJD will be recognized and diagnosed earlier in the disease process and that treatments can begin to be implemented that slow or potentially reverse disease progression.

## References

[1] Groveman BR, Foliaki ST, Orru CD, Zanusso G, Carroll JA, Race B, Haigh CL (2019). Sporadic Creutzfeldt-Jakob disease prion infection of human cerebral organoids. Acta Neuropathol Commun.

[2] Rey NL-G, Quiroga-Varela AQ, Garbayo E (2018). Advances in Parkinson’s disease: 200 years later. Front Neuroanat.

[3] Walga TK (2019). Understanding the experience and perspectives of Parkinson’s disease patient’s caregivers. Rehab Res Prac.

[4] Cerasa A, Koch G, Fasano A, Morgante F (2015). Levodopa-induced Dyskinesias in Parkinson’ Disease: Current Knowledge and Future Scenarios. Levodopa-induced Dyskinesias in Parkinson’ Disease: Current Knowledge and Future Scenarios.

[5] Zhang H, Lian Y, Ma Y, Chen Y, He C, Xie N, Wu C (2014). Two doses of botulinum toxin type A for the treatment of trigeminal neuralgia: observation of therapeutic effect from a randomized, double-blind, placebo-controlled trial. J Headache Pain.

[6] Lahiri D, Pattnaik S, Bhat A, Dubey S, Biswas A, Roy BK (2019). Young-onset sporadic Creutzfeldt-Jacob disease with atypical phenotypic features: a case report. J Med Case Rep.

[7] Hafez R (2015). Gamma knife radiosurgery for cerebellopontine angle vestibular schwannomas. Al-Azhar Assiut Med J.

[8] Lin EP, Crane BT (2017). The management and imaging of vestibular schwannomas. Am J Neuroradiol.

[9] Rus T, Lorber B, Trost M, Dobrecovic S, Caks JN, Popovic M, Kramberger MG (2018). High incidence of sporadic Creutzfeldt-Jacob disease in Slovenia in 2015: a case series. Dementia Geriatr Cogn Disord Extra.

[10] Espinosa PS, Bensalem-Owen MK, Fee DB (2018). Sporadic Creutzfeldt-Jakob disease presenting as nonconvulsive status epilepticus case report and review of the literature. Clin Neurol Neurosurg.

[11] Mead S, Rudge P (2017). CJD mimics and chameleons. Pract Neurol.

[12] Weils WA, Four SD, Seynaeve L, Flamezm A, Tousseyn T, Thal D, D’Haeseleer M (2018). Early-onset Creutzfeldt-Jacob disease mimicking immune-mediated encephalitis. Front Neurol.

[13] Manix M, Kalakoti P, Henry M, Thakur J, Menger R, Guthikonda B, Nanda A (2015). Creutzfeldt-Jacob disease: updated diagnostic criteria, treatment algorithm, and the utility of brain biopsy. Neurosurg Focus.

